# A Reliable Method to Recognize Soybean Seed Maturation Stages Based on Autofluorescence-Spectral Imaging Combined With Machine Learning Algorithms

**DOI:** 10.3389/fpls.2022.914287

**Published:** 2022-06-14

**Authors:** Thiago Barbosa Batista, Clíssia Barboza Mastrangelo, André Dantas de Medeiros, Ana Carolina Picinini Petronilio, Gustavo Roberto Fonseca de Oliveira, Isabela Lopes dos Santos, Carlos Alexandre Costa Crusciol, Edvaldo Aparecido Amaral da Silva

**Affiliations:** ^1^Department of Crop Science, College of Agricultural Sciences, São Paulo State University, Botucatu, Brazil; ^2^Laboratory of Radiobiology and Environment, Center for Nuclear Energy in Agriculture, University of São Paulo, Piracicaba, Brazil; ^3^Department of Agronomy, Federal University of Viçosa, Viçosa, Brazil

**Keywords:** seed maturity, seed quality, support vector machine, chlorophyll fluorescence, *Glycine max*

## Abstract

In recent years, technological innovations have allowed significant advances in the diagnosis of seed quality. Seeds with superior physiological quality are those with the highest level of physiological maturity and the integration of rapid and precise methods to separate them contributes to better performance in the field. Autofluorescence-spectral imaging is an innovative technique based on fluorescence signals from fluorophores present in seed tissues, which have biological implications for seed quality. Thus, through this technique, it would be possible to classify seeds in different maturation stages. To test this, we produced plants of a commercial cultivar (MG/BR 46 “Conquista”) and collected the seeds at five reproductive (R) stages: R7.1 (beginning of maturity), R7.2 (mass maturity), R7.3 (seed disconnected from the mother plant), R8 (harvest point), and R9 (final maturity). Autofluorescence signals were extracted from images captured at different excitation/emission combinations. In parallel, we investigated physical parameters, germination, vigor and the dynamics of pigments in seeds from different maturation stages. To verify the accuracy in predicting the seed maturation stages based on autofluorescence-spectral imaging, we created machine learning models based on three algorithms: (i) random forest, (ii) neural network, and (iii) support vector machine. Here, we reported the unprecedented use of the autofluorescence-spectral technique to classify the maturation stages of soybean seeds, especially using the excitation/emission combination of chlorophyll *a* (660/700 nm) and *b* (405/600 nm). Taken together, the machine learning algorithms showed high performance segmenting the different stages of seed maturation. In summary, our results demonstrated that the maturation stages of soybean seeds have their autofluorescence-spectral identity in the wavelengths of chlorophylls, which allows the use of this technique as a marker of seed maturity and superior physiological quality.

## Introduction

Soybean [*Glycine max* (L) Merrill] seeds with superior physiological quality (i.e., standard germination, greater vigor, and longevity) ensure adequate plant stand in the field, which significantly contributes to increased production (Finch-Savage and Bassel, 2016; Ebone et al., 2020). The stage of maturity at harvest is the primary factor that influences seed vigor (Finch-Savage and Bassel, 2016) because along the development, the physiological quality is built up to an ideal point of maturity, which is the point of greater vigor and longevity. In soybean seeds, germination, vigor and longevity begin to be acquired at maturation and are fully installed at late maturation (Lima et al., 2017; Basso et al., 2018).

In this legume, the reproductive (R) growth stages are characterized by flowering (R1 and R2), pod development (R3 and R4), seed development (R5 and R6), maturity and defoliation (R7, R8, and R9). Seeds begin to show germination and vigor in the dry stage from the R7 stage which is subdivided into three phases from R7.1 to R7.3. The R7.1 phase is marked by rapid accumulation of seed reserves and the beginning of physiological maturity (i.e., the acquisition of physiological quality starts), but most seeds at this stage are not able to tolerate desiccation, which negatively influences their germination (Lima et al., 2017). At the R7.2 stage, seeds reach mass maturity (time at which reserve deposition ceases), and they begin to disconnect from the mother plant. At this stage, seeds are more tolerant to desiccation compared to the R7.1 stage. The R7.1 and R7.2 stages belong to the maturation phase. When the seeds are completely disconnected from the mother plant, they reach the R7.3 stage, which is characterized by an intense degradation of chlorophylls and a progressive increase in the life span (longevity). From this stage, the late seed maturation phase begins. This increase continues through the stages of harvest point (R8) and final maturity (R9) (Leprince et al., 2017; Lima et al., 2017; Basso et al., 2018). Longevity is the physiological quality attribute that plays an important role in keeping the seeds viable during storage and preserving their vigor (Basso et al., 2018). Thus, mature soybean seeds from the last stages present greater basic properties, i.e., germination capacity, vigor, and longevity, insuring seed lots with superior physiological quality.

In this context, the development of markers to assess seed maturation stages is particularly important for seed companies in their internal seed quality control. This information may be used to detect the presence of immature seeds in the seed lots. Nowadays, visual inspection is the most applied method to diagnose immature soybean seeds. Visual inspection is based on morphological descriptors such as the common greening in the R7.1 and R7.2 stages. Another possibility to diagnose immature seeds is using the tetrazolium test (França-Neto and Krzyzanowski, 2020). In general, these tests are time-consuming and subjective, as they rely on the interpretation of different seed analysts, which reduces the reliability of the analysis.

A reliable and rapid alternative to classify soybean seeds at different stages of maturation would be through the development of image-based markers. New methodologies have been explored to monitor the physiological quality of seeds using images obtained through robust optical sensors (Galletti et al., 2020) to obtain more accurate results. For instance, the use of autofluorescence-spectral imaging technology has emerged as a strong tool for rapid diagnosis of seeds with superior physiological quality (Barboza da Silva et al., 2021a). This technique allows obtaining a high-resolution optical spectrum for each image pixel and produces a set of images of the same object for specific wavelengths. The spectral autofluorescence is emitted by different fluorophores that are naturally present in plant tissues (García-Plazaola et al., 2015). When these compounds are excited by ultraviolet-visible radiation of suitable wavelengths, they emit fluorescence, which is called autofluorescence, an intrinsic property of cells (Talamond et al., 2015).

Fluorophores have important biological implications for seed maturation and physiological quality. For instance, the high chlorophyll content (greenish seeds) reduces the seed lifespan (Zinsmeister et al., 2016). Galletti et al. (2020) demonstrated that immature carrot and tomato seeds emit high chlorophyll fluorescence, and these seeds had lower physiological quality compared to seeds with lower fluorescence. *Brassica oleracea* seeds with high chlorophyll fluorescence show a low germination rate (Jalink et al., 1998). These reports corroborate the negative effect of high chlorophyll accumulation on seed physiological quality. Nevertheless, lignin is another fluorophore with positive implications for physiological quality, as reported by Barboza da Silva et al. (2021a) in soybean seeds. Hence, the association of these compounds with their respective wavelengths using autofluorescence-spectral images makes it possible to categorize numerous phenomena related to the physiological quality of seeds and simplify the obtaining of analyses and information.

The information generated from multispectral images can be interpreted with artificial intelligence (Fonseca de Oliveiran et al., 2022). This is possible using machine learning algorithms [e.g., random forest (RF), neural network (NN), and support vector machine (SVM)] on the data extracted from the multispectral images. These algorithms allows verify the accuracy of the method used and have enabled a better characterization of the physical and physiological quality of soybean seeds (Momin et al., 2017; Mahajan et al., 2018; Lin et al., 2019; Barboza da Silva et al., 2021a). Beside this, the use of machine learning algorithms on data extracted from multispectral images demonstrates that, through artificial intelligence, it is possible to interpret the results in real time, and thus provide a future automation of the analysis process in the seed industry. With these technologies, most of the limitations now faced by traditional methods based on visual seed inspection can be overcome (Medeiros et al., 2020).

The autofluorescence-spectral imaging technique combined with machine learning algorithms can be interesting to classify the maturation stages of soybean seeds, as this method is based on the detection of fluorophore signals present in the seeds, which change during maturation (Teixeira et al., 2016; Lima et al., 2017). Here, we investigated autofluorescence-spectral identity patterns in the final stages of soybean seeds (i.e., R7.1, R7.2, R7.3, R8, and R9) together with machine learning models. From this knowledge, we developed a reliable method that could be potentially used by the seed industry to screen the maturation stages and associate them with the physiological quality of commercial seed lots.

## Materials and Methods

### Seed Material

Seeds of the commercial cultivar MG/BR 46 “Conquista” were propagated in a greenhouse in 11 L pots filled with sandy textured soil (778 g.kg^–1^ of sand). Five seeds were sown per pot (*n* = 95), and after seedling emergence, two plants per pot were kept, and the others were eliminated (i.e., 190 plants were produced). The humidity of the pots was kept close to the field capacity (previously determined) and was obtained by means of a humidity sensor, positioned at a depth of 10 cm, twice a day (10:00 a.m. and 16:00 p.m). If a difference was detected during reading, the corresponding volume of water was added to return the soil to field capacity. The air temperature average during the experiment was maintained at 24.2°C and the relative humidity at 65%.

During the maturation phase, the pods were manually collected according to the descriptions in Table 1, and the seeds classified according to their respective stage, i.e., R7.1, R7.2, R7.3, R8, and R9, as initially described by Fehr and Caviness (1977) with the adaptations proposed by Lima et al. (2017) and Basso et al. (2018) (Table 1).

**TABLE 1:** Description of soybean seed maturation stages, and the corresponding seed moisture content and morphological characteristics of pods and seeds used in this study.

Phase	Stage	Description	Seed moisture content (gH_2_O per g.DW)	Morphology	
				Pod	Seeds
Maturation	R7.1	Beginning of maturity	*1.56 ± 0.002	Completely green	Green with prominent yellow embryo
	R7.2	Mass maturity	1.26 ± 0.01	Yellow with green spots	Yellow with greenish spots in the central region
Late maturation	R7.3	Seed disconnected from mother plant	1.17 ± 0.01	Completely yellow	Completely yellow
	R8	Harvest point	0.46 ± 0.02	Yellow with brow spots	Yellow with opaque tegument and rubbery consistency
	R9	Final maturity	0.11 ± 0.004	Completely brow	Yellow with opaque seed coat and dry

*Mean of two replicates of five seeds ± standard deviation. DW, Dry weight.

Immediately after fruit collection, the seeds were extracted manually, and immature seeds were subjected to rapid drying to prevent stage advancement. For this, they were distributed in single layers on a metallic screen suspended inside a plastic box containing silica gel at the bottom (relative humidity at 15%) and incubated at 20°C. Successive weighing of the samples was carried out until they reached 10% of moisture content on a wet basis, monitored through a drying curve. The drying period depended on the initial moisture content of the seeds in each developing stage. However, this time did not exceed 60 h for each seed sample. The moisture content was measured by the oven method (ISTA, 2020), before and after drying. The silica gel was constantly changed to guarantee a constant 15% relative humidity inside the drying environment. The dried seeds were stored at 10°C and 55% relative humidity until the beginning of the experiment, a period not exceeding 30 days.

### Autofluorescence-Spectral Imaging and Data Extraction

Multispectral images were captured from four replicates of 25 seeds using a VideometerLab4™ instrument (Videometer A/S, Herlev, Denmark). This system can capture high resolution autofluorescence-spectral images (2192 × 2192 pixels) using light-emitting diodes at different excitation wavelengths combined with optical filters (long-pass filters). The seeds were placed on an acetate sheet (5.0 cm × 8.5 cm) in the same position (hilum to the left) using double-sided adhesive tape. Before image acquisition, the light setting was adjusted to optimize the strobe time of each illumination type, resulting in a better signal-to-noise ratio such that the captured images could be directly comparable. Light setup was calibrated using a representative sample and saved for all subsequent images. Autofluorescence-spectral images of each sample were generated in one sequence during 1 min using different excitation/emission combinations: 365/400, 405/500, 430/500, 450/500, 470/500, 405/600, 515/600, 540/600, 570/600, 630/700, 645/700, and 660/700 nm. Autofluorescence data were extracted using VideometerLab™ software (version 3.14.9). For this, a segmentation image technique based on thresholding was applied to separate the seeds from the background, which was represented by zero. In the segmented seeds, a normalized canonical discriminant analysis (nCDA) algorithm was applied to highlight the autofluorescence signals pixel-to-pixel for each excitation/emission combination. The nCDA algorithm uses a 10% trimmed mean, eliminating the influence of outliers (the lowest 10% and the highest 10% of the data) (Barboza da Silva et al., 2021b), transforming grayscale images into score images with red-green-blue color codes (Bianchini et al., 2021). The pixel values in the autofluorescence images depend on the concentration of fluorophores. RGB images were also acquired using the same sensor. They were captured to compare seed characteristics that are not visible to the naked eye with autofluorescence-spectral images.

### Dry Weight and Seed Area

Four replicates of 15 seeds from each maturation stage were dried in an oven with forced air circulation at 60°C for 72 h, and then the samples were weighed with an analytical scale. Seed area measurement was performed by using the autofluorescence images captured as previously described. Data were extracted using the binary large object (blob) toolbox of the VideometerLab™ software (version 3.14.9); each blob was represented as a soybean seed.

### Germination Test

Four replicates of 25 seeds were germinated on paper towels moistened with deionized water 2.5 times the mass of the dry paper. The rolled paper towels were kept at 25°C in the dark (ISTA, 2020). The percentage of normal seedlings according to the ISTA (2020) was recorded on the 3rd and 5th days after sowing.

### Vigor Tests

The accelerated aging test was performed using four replicates of 25 seeds arranged in a single layer on a wire mesh screen suspended inside a plastic box (3.5 cm × 11.0 cm × 11.0 cm) containing 40 mL of water. The boxes were sealed and kept at 41°C for 48 h. After this period, the seeds were sown as described for the germination test, and normal seedlings were recorded at 3 days after sowing. To evaluate the seedling growth, the seeds were artificially aged for 48 h as described above, and then 10 seeds were distributed alternately on the upper third of the surface of the moistened paper towels. The towels were rolled up and incubated at 25°C in the dark for 7 days. The hypocotyl, root, and total seedling length were measured in the normal seedlings. The seedling vigor index (SVI) was calculated as proposed by Egli and TeKrony (1979) and adapted by Ribeiro-Oliveira et al. (2021) using the algebraic expression: $S\,V\,I=\frac{A\,A}{N\,S}\,100$, where: *SVI* is expressed in percentage, *AA* is the number of normal seedlings counted from the aged seeds and *NS* is the number of normal seedlings counted in the germination test on the 5th day.

### Chlorophyll *a, b* and Total Carotenoids

Four samples of 10 g of seeds were ground until obtaining a fine homogeneous powder. 10 mL of 80% acetone was added to four replicates of 2 g of each sample (collected from the macerate mentioned earlier). Following this, the solution was mixed using a vortex, and after 30 min, the aqueous suspension was filtered. Total carotenoids, chlorophyll *a* and chlorophyll *b* were determined by the absorbance at 470, 645, and 663 nm, respectively (Arnon, 1949; Reis et al., 2019). All steps were performed in the dark and with the use of green light, when necessary.

### Classification of Maturation Stages Using Machine Learning Algorithms

We created machine learning models using autofluorescence-spectral data extracted from each individual seed from R7.1, R7.2, R7.3, R8, and R9 stages (*n* = 500 seeds). The models used on these data were based on NN (solver: Stochastic Gradient Descent; hidden layer sizes: two layers with 25 neurons in each; Activation function: Tanh; Learning rate: adaptive; maximum number of interactions: 5000), SVM and RF algorithms. Data from 350 seeds were used for internal validation in supervised training (K-fold = 5) and 150 seeds were used for external validation.

### Statistical Design

**FIGURE 1:**
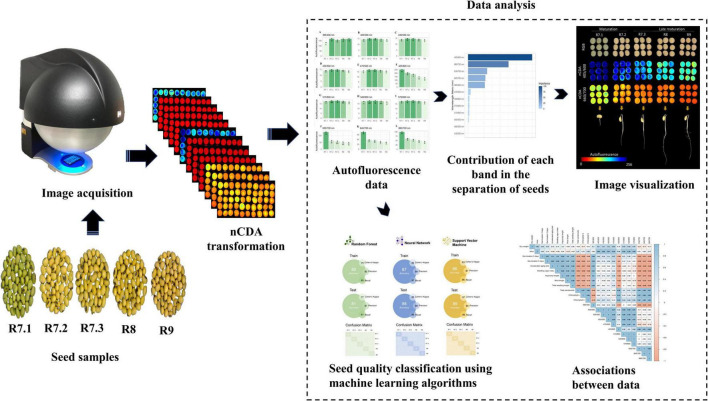
Flowchart of the main steps to classify the maturation stages of soybean seeds based on autofluorescence-spectral imaging combined with machine learning algorithms.

Figure 1 shows the main procedures for obtaining and analyzing the data. Autofluorescence-spectral data, physical and physiological properties, and pigment content were analyzed using analysis of variance (ANOVA). When significant, the means (*n* = 20) were compared by Tukey’s test (*P* < 0.05). The RF algorithm using autofluorescence-spectral data was performed to select the most import bands to segment the stages. We created machine learning models based on the NN, RF, and SVM algorithms, and the performance of the models was evaluated using four metrics: accuracy, Cohen’s Kappa coefficient, precision, and recall. All metrics were calculated using a confusion matrix. The Pearson’s correlation coefficient was measured to investigate the relationship between excitation/emission combination and physical and physiological properties, and pigment content. All analyzes were performed using the R 4.0.0 software (R Core Team, 2021).

## Results

### Changes During Seed Maturation

**FIGURE 2:**
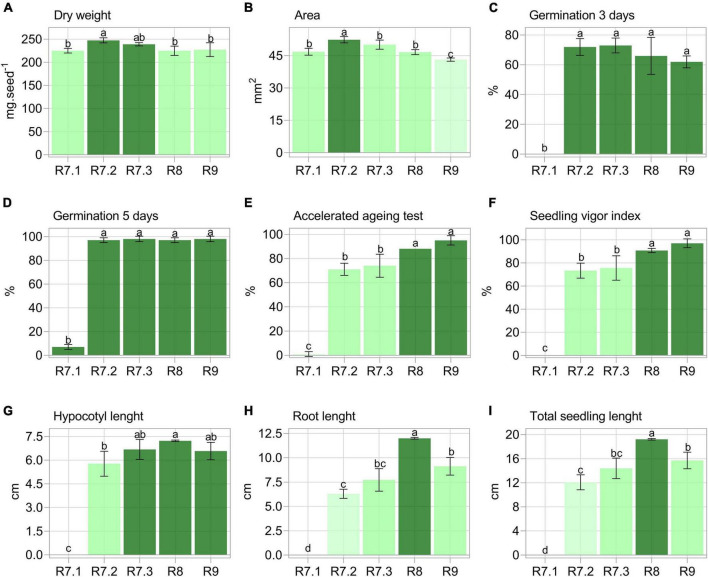
Physical and physiological properties of soybean seeds from different maturation stages. Each bar represents the mean of four replicates of 25 seeds ± standard deviation. Different letters indicate significant difference (α = 0.05) by Tukey test (*n* = 20).

The highest mass accumulation was achieved in the R7.2 stage (Figure 2A). Seed area was maximum in the R7.2 stage and decreased as maturation progressed (Figure 2B). Seeds showed maximum germination potential (> 97%) in the R7.2 stage, maintaining it until final late maturation (R9) (Figures 2C,D). However, when seeds were artificially aged, they only reached the highest germination and SVI in the R8 and R9 stages (Figures 2E,F). Regarding seedling growth, two results were important: (i) greater elongation of the hypocotyl in seedlings produced from seeds in the R8 stage in contrast to R7.2; and (ii) greater root length and total seedling elongation for the R8 stage (Figures 2G–I).

**FIGURE 3:**
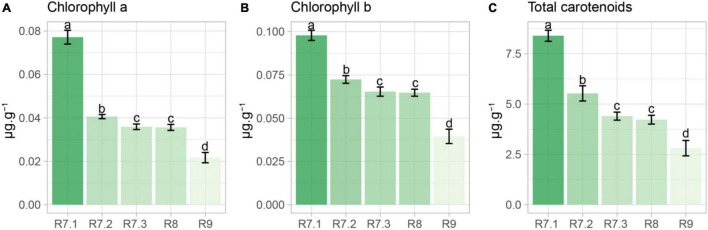
Chlorophyll *a*, chlorophyll *b* and total carotenoids in soybean seeds from different maturation stages. Each bar represents the mean of four replicates ± standard deviation. Different letters indicate significant difference (α = 0.05) by Tukey test (*n* = 20).

The contents of chlorophyll *a*, chlorophyll *b* and total carotenoids progressively reduced along maturation, with the lowest values verified in seeds in the R9 stage (final maturity). The content of these pigments did not change from R7.3 to R8 stage (Figure 3).

### Segmentation of Maturation Stages Using Autofluorescence-Spectral Imaging

**FIGURE 4:**
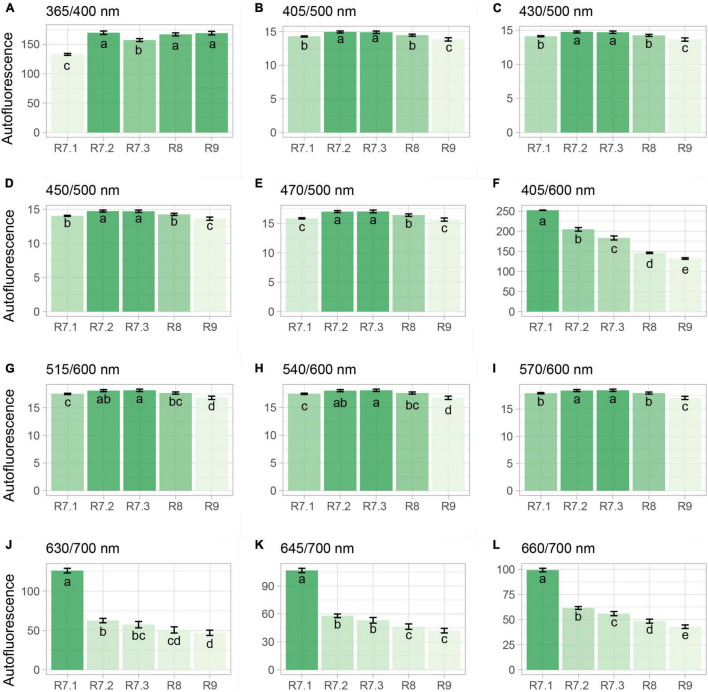
Autofluorescence-spectral data from different excitation/emission combinations in soybean seeds at different seed maturation stages. Each bar represents the mean of four replicates of 25 seeds ± standard deviation. Different letters indicate significant difference (α = 0.05) by Tukey test (*n* = 20).

The 365/400 nm excitation/emission combination was not capable of separating all the seeds in the late maturation phase from those that had recently reached the point of mass maturity, i.e., R7.2 stage; however, this combination efficiently segmented the R7.1 stage due to its lower fluorescence (Figure 4A). The combinations of 405/500, 430/500, 450/500, 515/600, 540/600, and 570/600 nm successfully segmented the R9 stage from the others (Figures 4B–E,G–I). Finally, the combinations of 405/600 and 660/700 nm perfectly segmented the five maturation stages in descending order from R7.1 to R9 stage (i.e., beginning of maturation to final late maturation) (Figures 4F,L). Using the combination of 630/700 and 645/700 nm there was a separation of the late maturation (R8 and R9) from the early maturation phases (R7.1 and R7.2) (Figures 4J,K).

**FIGURE 5:**
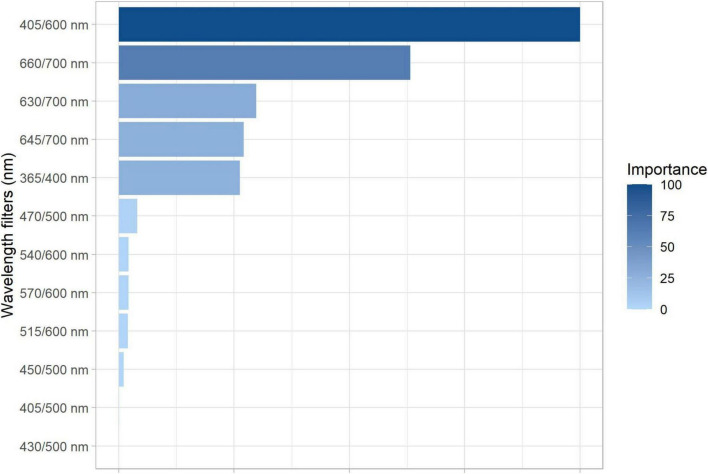
Gini-based importance for each excitation/emission combinations in the random forest analysis to segment soybean seeds of different maturation stages: R7.1, R7.2, R7.3, R8, and R9 (*n* = 500 seeds).

The RF algorithm based on Gini coefficient was applied to test the importance of each excitation/emission combination in the segmentation of the maturation stages (i.e., R7.1, R7.2, R7.3, R8, and R9) using the autofluorescence-spectral data. From this, it was noted that the combination of 405/600 nm has greater importance in the separation of the groups according to their maturation level; followed by the combination of 660/700 nm (Figure 5).

**FIGURE 6:**
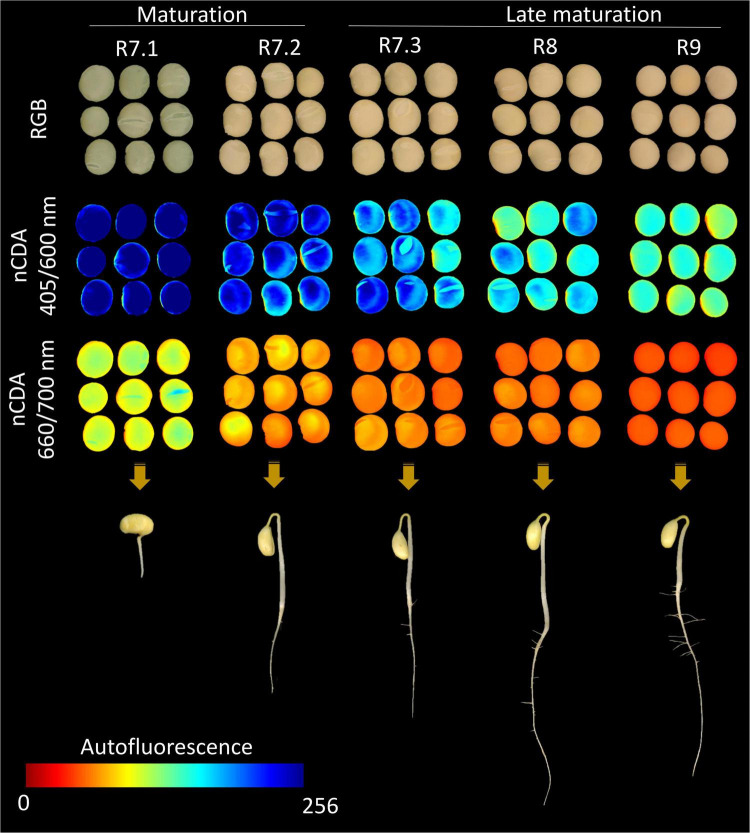
Framework of soybean seeds at different maturation stages assisted by RGB images (each individual pixel is represented by red, green and blue channels) and corresponding autofluorescence-spectral images captured in the excitation/emission combinations of 405/600 nm (chlorophyll *b*) and 660/700 nm (chlorophyll *a*), after image transformation by the nCDA algorithm. The seedling images were captured at 7 days after sowing using a representative seedling of each maturation stage.

We constructed a maturation progress framework using RGB and autofluorescence-spectral images acquired at 405/600 and 660/700 nm (Figure 6), as these combinations perfectly separated the seeds of each maturation stage (Figures 4F,L). These combinations were identified as the most important for this identification (Figure 5). The grayscale images were transformed by the nCDA algorithm, and autofluorescence signals were displayed using a color scale ranging from zero (red) to 256 (blue) with pixel-to-pixel mapping, wherein increasing intensity indicates greater autofluorescence. It was noticed that after the R7.2 stage, the RGB images were similar between maturation stages, which would lead to subjective analysis if purely visual. At the same time, the autofluorescence images in the combinations of 405/600 and 660/700 nm exhibited a progressive reduction in fluorescence signals (reduction in pixel values) as maturation progressed (Figure 6), in agreement with the previous description (Figures 4F,L). Additionally, we illustrated that as autofluorescence signals decreased throughout maturation, the ability of seedlings to establish increased, especially in late maturation (Figure 6).

### Maturation Stage Classification Based on Different Machine Learning Algorithms

**FIGURE 7:**
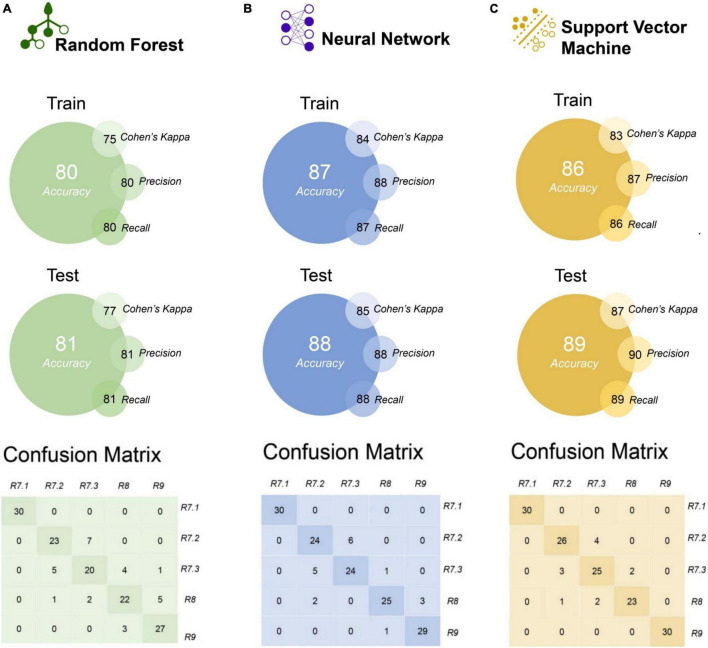
Comparison of the random forest (RF), neural network (NN), and support vector machine (SVM) algorithms discriminating soybean seed maturation stages using autofluorescence-spectral data, and performance of the models based on accuracy, kappa, precision and recall (presented in percentage). The models were created using autofluorescence-spectral data extracted from seeds in the R7.1, R7.2, R7.3, R8, and R9 stages (*n* = 500 seeds).

The RF, NN, and SVM algorithms showed considerable performance in the separation of soybean maturation stages based on the autofluorescence-spectral data (Figure 7). The NN and SVM algorithms showed higher accuracy (≥ 86%) for training and external test validation, but the SVM algorithm showed slightly greater performance for all metrics on external test validation sets: accuracy (89%), Cohen’s Kappa (87%), precision (90%), and recall (89%) (Figure 7C). The confusion matrix demonstrates the recognition of the maturation stages by each algorithm, with a higher hit rate achieved by NN and SVM in relation to RF algorithm (Figure 7). Nevertheless, all algorithms achieved excellent performance in classifying seeds in the R7.1 stage (100% for all algorithms). In addition, SVM can perfectly distinguish the R9 stage (final maturity) from the others (Figure 7C).

### Correlations Between Physical and Physiological Parameters, Pigment Content, and Autofluorescence-Spectral Markers During Seed Maturation

**FIGURE 8:**
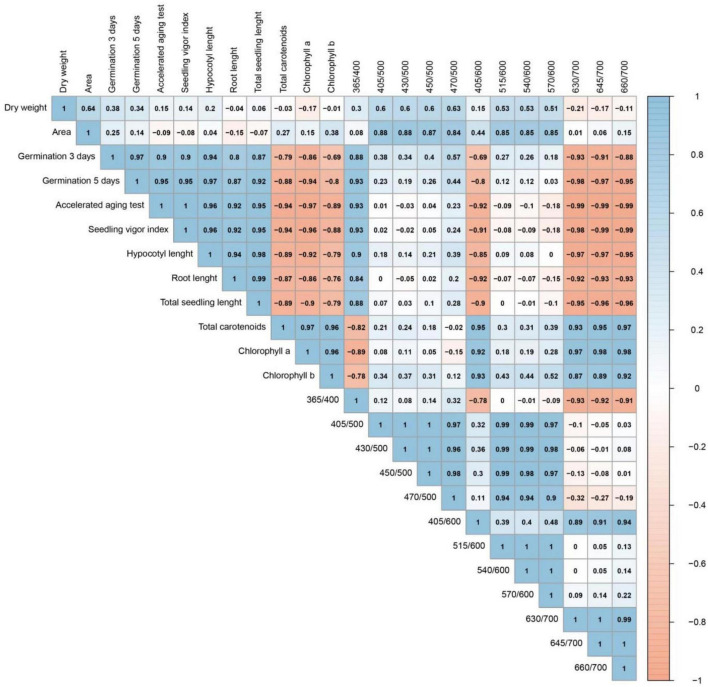
Pearson’s correlation coefficients between physical properties, physiological quality, pigment content and autofluorescence-spectral markers (nm) in soybean seeds of different maturation stages (R7.1, R7.2, R7.3, R8, and R9).

The highest coefficient correlations (Figure 8) were obtained between 630/700 nm (or 645/700 or 660/700 nm) and the accelerated aging test (−0.99), and between 645/700 nm (or 660/700 nm) and the SVI (−0.99). Other high coefficient correlations should be highlighted: 630/700 nm vs. either germination 5 days (−0.98), SVI (−0.98), hypocotyl length (−0.97), chlorophyll *a* content (0.97); 645/700 nm vs. either chlorophyll *a* content (0.98), germination 5 days (−0.97), hypocotyl length (−0.97); 660/700 nm vs. either chlorophyll *a* content (0.98), total carotenoids (0.97). For seed physical properties, the highest coefficients were obtained between 405/500 nm (or 430/500 nm) vs. seed area (0.88) (Figure 8).

## Discussion

The use of seeds with superior physiological quality has contributed to the adequate establishment of the seedlings under field conditions. Mature seeds have better properties to achieve this aim. However, recognizing the identity of seed maturation stages using the current techniques is a time-consuming and subjective task. In this sense, here we present a robust way to classify soybean seeds at different stages of maturation using autofluorescence-spectral of the seeds combined with machine learning algorithms.

Firstly, we found that the physiological changes during maturation (Figure 2) were similar to those previously reported by Lima et al. (2017) and Basso et al. (2018) for soybean seeds, which allowed us to explain some important results. First, seeds from the R7.1 stage are unable to tolerate desiccation (Lima et al., 2017), which results in their low germination, stress resistance, and seedling organ elongation capacity (Figure 2). Desiccation tolerance is acquired between R7.1 and R7.2 stages (Basso et al., 2018), and explains the abrupt increase in germination at the mass maturity stage (R7.2), which remains until the end of seed development (late maturation) (Figures 2C,D). Although soybean seeds are able to germinate upon reaching mass maturity (Figure 2A), they still need to go through the late maturation phase, which starts at the R7.3 stage. This is necessary for increasing storage capacity (Lima et al., 2017), as demonstrated here by its analogous test (i.e., the accelerated aging test) (Batista et al., 2021), and for the elongation of seedling organs (Basso et al., 2018), as shown in our results (Figures 2E–I).

As late maturation progresses, the seeds gain greater physiological dissimilarity from those in the mass maturity stage, i.e., R7.2 (Figure 2). However, the R7.3 stage, which delimits the beginning of late maturation, is difficult to separate from R7.2, which is due to the overlap of events that define the transition from maturation to late maturation (Lima et al., 2017). This difficulty was supported by our results (Figure 2).

From the use of the autofluorescence-spectral imaging, we found a marked reduction in autofluorescence along the maturation process by using the excitation/emission combinations of 405/600, 630/700, 645/700, and 660/700 nm (Figures 4F,J–L). These combinations allowed us to measure chlorophyll fluorescence signals (Galletti et al., 2020; Barboza da Silva et al., 2021a; Oliveira et al., 2021). These pigments degrade intensely in the late maturation stages of leguminous seeds (Leprince et al., 2017); however, the chlorophyll degradation starts earlier as corroborated by our results of chlorophyll *a* and *b* content throughout maturation (Figures 3A,B). These results were determined after we found that the wavelengths for segmentation of maturation stages coincided with the chlorophyll excitation bands.

Thus, in parallel with the reduction of chlorophylls as maturation progressed, the autofluorescence intensity at 405/600, 630/700, 645/700, and 660/700 nm decreased (Figures 4F,J–L). It was obtained better results at 405/600 and 660/700 nm (Figures 4F,L), which are bandwidths directly related to excitation/emission of chlorophyll *b* and *a*, respectively; the visualization of this process is clear in the autofluorescence images (Figure 6). We would also like to emphasize that the results of the RF analysis made it clear that these excitation/emission combinations are the most important for the separation of the stages based on their autofluorescence-spectral in relation to the other combinations (Figure 5). Prominently, these bandwidths allowed us to segregate the R7.3 stage from the others in the late maturation phase (Figures 4F,L), unlike the direct analysis of chlorophylls based on conventional methods (Figures 3A,B). In addition, they separated the R8 and R9 stages (Figures 4F,L), which have a similar physiological identity (Figure 2). These results highlight the advantage of using chlorophyll autofluorescence signals to separate soybean seeds at different stages of maturation.

According to García-Plazaola et al. (2015), chlorophylls are fluorophores that have their fluorescence used as a non-invasive tool in the evaluation of plant photosynthesis. The technique is primarily used to monitor sensitivity to the various types of abiotic stresses that plants face as they develop (Shin et al., 2021). Recently, due to its sensitivity, speed and non-invasiveness, this technique has also been applied to control the physiological quality of seeds. However, contrary to the principle used in plants, chlorophyll fluorescence has generally negative implications in seeds as previously reported for immature carrot and tomato seeds (Galletti et al., 2020) and *Brassica oleracea* seeds (Jalink et al., 1998). These results agree with our findings (Figures 2, 4F, J–L). Therefore, extending this knowledge, we present the use of this technique as a sensitive and non-invasive tool to classify soybean seeds according to their degree of maturity, as it is associated with the process of chlorophyll degradation during maturation, while seeds acquire physiological quality. These new findings contribute to the use of chlorophyll fluorescence to solve applied problems, benefiting the seed industry in classifying seeds according to the stage of maturity.

As a consequence of our findings, it is possible to state that the excitation/emission combinations related to chlorophyll fluorescence (405/600, 630/700, 645/700, and 660/700 nm) demarcate the spectral identity of seed maturity in soybean (Figures 4F,J–L, 5). This knowledge has two important implications: (i) lower autofluorescence-spectral signals at these excitation/emission wavelengths may be associated with higher maturity seed lots; and (ii) the opposite, the high intensity of autofluorescence-spectral in these bandwidths indicates the presence of seeds with low physiological quality (i.e., those belonging to early maturity stages), which compromises their commercialization.

It is important to highlight that the reduction of fluorescence signals at the bandwidths of chlorophylls in parallel with the increase in seed vigor throughout maturation obtained strong negative correlations (> −0.9) between different excitation/emission combinations (405/600, 630/700, 645/700, and 660/700 nm) and physiological parameters related to seed vigor, such as the accelerated aging test, SVI and total seedling length (Figure 8). The artificial aging test is a reliable method to test the physiological quality of the seed lot and to predict when the seeds can reach the limit of viability (Fenollosa et al., 2020; Batista et al., 2021), which provides additional information for the conservation of the species’ germplasm (Hay and Whitehouse, 2017). Thus, this test is a strong tool for the seed industry and the germplasm banks management. The high correlation between the accelerated aging test and autofluorescence-spectral data reported in this study indicates that the use of this new technology offers, in relation to the artificial aging method, agility and greater reliability in the analysis of the degree of seed maturity. Furthermore, it is a non-invasive way to perform the analysis, which is important for germplasm banks.

We created machine learning models for verify the accuracy and automatic classification of maturation stages using RF, NN, and SVM algorithms. The models achieved high accuracy (≥ 80%) using the autofluorescence-spectral signals based on different excitation/emission combinations (Figure 7). These results demonstrate the potential to automate the process of classifying maturation stages of soybean seeds, providing information about the maturity of the lots in a robust way (Figure 7). Machine learning algorithms have been successfully used to improve the diagnosis of the physiological quality of soybean seeds (Medeiros et al., 2020; Barboza da Silva et al., 2021a), and now we advanced in this field demonstrating their predictive effectiveness in classifying seeds at different maturation stages.

We emphasize that the chlorophylls which fluoresce at the excitation/emission combinations of 405/600, 630/700, 645/700, and 660/700 nm are not associated with an increase in seed mass or a decrease in seed area (Figure 8). This highlights that the dynamics of these fluorophores is more pronounced than the morphological changes. Therefore, this technique provides greater reliability to classify soybean maturation stages.

We should highlight the separation of the R9 stage (i.e., final maturity) from the others by low fluorophore signals measured at the combinations of 405/500, 430/500, 450/500, 470/500, 515/600, 540/600, and 570/600 nm (Figures 4B–E,G–I). Fluorophore such as lignin, ferulic acid, coumarins, flavonoids, and phenols, among others, emit fluorescence in these bands (Donaldson, 2020). So far, these compounds do not have a clear role in the physiological quality of seeds. Despite this, Barboza da Silva et al. (2021a) associated superior physiological quality of soybean seeds with lower accumulation of phenols in the seed embryo. In part, this information helps us to explain the reasons for detecting low fluorescence signals at these bands in the final stage of maturity (the R9 stage), as these seeds had superior physiological quality (Figure 2) and possibly lower phenol content.

Regarding the other compounds that change in parallel to chlorophylls throughout maturation, such as lignin and phenolic compounds, we would like to point out that, as verified by Barboza da Silva et al. (2021a), they have no significant fluorescence in the same wavelength as the chlorophylls. Such information allows us to exclude possible interferences from the tegument lignification process and the presence of phenols in our results. Although some amount of these compounds can fluoresce in the excitation/emission combinations of chlorophylls, their value is negligible since these combinations do not provide their peak detection in soybean seeds.

Finally, based on the high predictive accuracy of the autofluorescence-spectral imaging in the discrimination of the soybean seed maturation stages (Figure 7) and strong coefficient correlations between certain excitation/emission combinations and the physiological quality of the seeds (Figure 8), we confirm the validity of our initial idea. This is especially true when we see the results of the chlorophyll fluorescence signals from the 405/600 nm and 660/700 nm excitation/emission combinations (Figures 4F,L, 5, 6).

### Perspectives

With the knowledge generated in this research, we expect that autofluorescence-spectral imaging technology will become a potential tool in the seed industry. It offers a much more reliable way to classify the maturation stages in a non-invasive way. This is important since a higher degree of maturation positively impacts the establishment of seedlings in the field and seed production.

Here, we found that this emerging methodology (i.e., autofluorescence-spectral) provides rapid and accurate results in identifying seed maturity using the excitation/emission combinations of 405/600, and 660/700 nm that are highly correlated with physiological parameters and pigment content, particularly chlorophylls. Therefore, this can open new research possibilities toward finding chlorophyll fluorescence markers using different genotypes. There may be some divergence in the chlorophyll content inherent in each material that may result in specific fluorescence patterns.

The insights gained from this research can also contribute to easily detect the “green seed problem” that occurs due to climate changes (high temperatures and drought stress). This is characterized by chlorophyll retention in mature seeds, and is associated with lower seed quality. The possibility of detecting this problem using chlorophyll fluorescence deserves to be validated in future research.

Another interesting point is related to the strong coefficient correlations (*r* > 0.8) between the excitation/emission combination of 365/400 nm and the physiological quality acquired by seeds during maturation (Figure 8), although this combination had low specificity in separating the seed maturation stages (Figure 4A). Considering that the combination of 365/400 nm has already been recommended to classify soybean seeds with superior physiological quality (Barboza da Silva et al., 2021a), our results add the information that low fluorescence signals in this combination are associated with the presence of immature seeds (low viability), which are unsuitable to seed lots.

Finally, the assertiveness of the machine learning models offers an opportunity to automate different phases of seed production. For instance, the incorporation of the autofluorescence-spectral imaging technique into the soybean seed processing phase can be useful to anticipate the diagnosis of seeds with high chlorophyll content (i.e., seeds with low physiological potential). This automation would reduce the subjective measurements from tests that rely on the human eye.

## Conclusion

The use of machine learning models based on the autofluorescence-spectral imaging of soybean seeds allows the selection of seeds according to their maturation stage, especially using the excitation/emission combinations of 405/600 and 660/700 nm.

## Data Availability Statement

The raw data supporting the conclusions of this article will be made available by the authors, without undue reservation.

## Author Contributions

TB conceived research ideas and wrote the first draft of the manuscript. TB, CM, and EA contributed to design of the study. TB, AP, GF, and IS conducted the experiments. CC supported the production of the seeds. TB and AP organized the database. AM performed the statistical analysis. TB, CM, and GF wrote sections of the manuscript. EA supervised the research. All authors contributed to manuscript revision, read, and approved the submitted version.

## Conflict of Interest

The authors declare that the research was conducted in the absence of any commercial or financial relationships that could be construed as a potential conflict of interest.

## Publisher’s Note

All claims expressed in this article are solely those of the authors and do not necessarily represent those of their affiliated organizations, or those of the publisher, the editors and the reviewers. Any product that may be evaluated in this article, or claim that may be made by its manufacturer, is not guaranteed or endorsed by the publisher.
